# Perforated hepatic flexure adenocarcinoma mimicking pyogenic hepatic abscess: A CT diagnostic pitfall

**DOI:** 10.1016/j.radcr.2026.10.032

**Published:** 2026-11-07

**Authors:** Getnet Bimer Kelemu, Dress Enyew Mhret, Yeshiambel Gete Tsegaw, Shimalis Fayisa

**Affiliations:** a Samera University, Afar, Ethiopia; b Kasma Diagnostic Center, Bahir Dar, Ethiopia; c College of Medicine and Health Sciences, Bahir Dar University, Bahir Dar, Ethiopia; d Care Land General Hospital, Addis Ababa, Ethiopia

**Keywords:** Colon carcinoma, Hepatic abscess, Perforated colon cancer, Computed tomography, Hepatic flexure adenocarcinoma

## Abstract

Perforated colorectal carcinoma may present with inflammatory and septic features that obscure the underlying malignancy. Tumors arising at the hepatic flexure are particularly challenging because of their close anatomical relationship with the liver, allowing perforation-related inflammation and necrotic hepatic metastases to mimic pyogenic hepatic abscesses on imaging. A 58-year-old man with hypertension and human immunodeficiency virus (HIV) infection receiving antiretroviral therapy presented with acute abdominal pain, fever, chills, and systemic inflammatory symptoms. Ultrasound demonstrated hepatic lesions and irregular hepatic flexure wall thickening suspicious for malignancy, while contrast-enhanced CT showed multiple abscess-like hepatic lesions with features of both metastatic disease and perforation-related inflammation. Emergency laparotomy revealed a perforated hepatic flexure tumor densely adherent to the liver, with fibrinopurulent exudate, an inflammatory tract between the hepatic flexure and adjacent liver, multiple hepatic nodules, and peritoneal tumor seeding; histopathology confirmed moderately differentiated adenocarcinoma with hepatic and nodal metastases and central necrosis in the hepatic lesions (pT4N2M1). This case highlights the diagnostic overlap between pyogenic hepatic abscesses and necrotic hepatic metastases associated with perforated colorectal carcinoma. When abscess-like hepatic lesions coexist with irregular colonic wall thickening, regional lymphadenopathy, or loss of normal tissue planes, colorectal malignancy should remain an important consideration despite accompanying inflammatory features.

## Introduction

Colorectal carcinoma is among the leading causes of cancer-related morbidity and mortality worldwide [1,2]. Typical presentations include altered bowel habits, lower gastrointestinal bleeding, anemia, weight loss, or intestinal obstruction. However, perforated colonic carcinoma may occasionally present with atypical inflammatory manifestations that obscure the underlying diagnosis [3,4]. The combination of perforated hepatic flexure adenocarcinoma, abscess-like hepatic lesions, and suspected hepatocolic communication represents an unusual diagnostic challenge.

Tumors located at the hepatic flexure pose a unique diagnostic challenge because of their close anatomical relationship with the liver and surrounding peritoneal structures. Local tumor perforation and inflammatory extension may result in reactive inflammatory changes, hepatic invasion, or fistulous communication with adjacent hepatic tissue [4,5]. In rare cases, these findings may radiologically mimic pyogenic hepatic abscesses [5,6].

Distinguishing pyogenic hepatic abscesses from necrotic metastatic lesions or direct tumor invasion is clinically important because the therapeutic approach and prognosis differ substantially between infectious and malignant processes [7]. Imaging findings such as central necrosis, peripheral rim enhancement, and adjacent inflammatory changes may result in considerable diagnostic overlap [8,9]. This diagnostic challenge may be particularly relevant in HIV-positive patients, in whom infectious etiologies may initially be favored, potentially delaying recognition of an underlying malignancy [10].

We report a case of perforated hepatic flexure adenocarcinoma in an HIV-positive patient that radiologically mimicked multiple pyogenic hepatic abscesses on contrast-enhanced computed tomography.

## Case presentation

A 58-year-old man with a history of hypertension on amlodipine and human immunodeficiency virus (HIV) infection on long-term antiretroviral therapy presented with acute onset epigastric and right upper quadrant abdominal pain. The pain was associated with fever, chills, rigors, anorexia, nausea, headache, and generalized diaphoresis. He reported good adherence to antiretroviral therapy, and no known opportunistic infections were documented. Recent CD4 count and viral load measurements were unavailable.

On examination, the patient appeared acutely ill and diaphoretic. Vital signs were as follows: temperature, 38.5°C; pulse rate, 102 beats per minute; respiratory rate, 20 breaths per minute; and blood pressure, 100/80 mmHg. Abdominal examination demonstrated tenderness in the epigastric, right upper quadrant, and right lower quadrant regions without peritoneal guarding.

Laboratory investigations showed relative neutrophilia (83.6%) without marked leukocytosis (white blood cell count, 9.9 × 10³/μL). Hemoglobin level was 11.4 g/dL. Liver function tests, serum amylase, and lipase levels were within normal limits.

Abdominal ultrasound demonstrated multiple hepatic lesions with mixed echogenicity and peripheral hypoechoic halos involving both hepatic lobes. In addition, there was focal asymmetric wall thickening at the hepatic flexure with adjacent pericolonic fat stranding. The initial sonographic impression favored colonic malignancy with hepatic metastases.

Contrast-enhanced CT of the abdomen demonstrated multiple well-defined hypoattenuating lesions in the right hepatic lobe and caudate lobe, with an attenuation value of approximately 20 HU (Fig. 1). The lesions demonstrated thin, smooth peripheral enhancement with central nonenhancing components. The largest lesion measured approximately 5.7 × 5.8 cm in segment VII (Fig. 2).

**Fig. 1:**
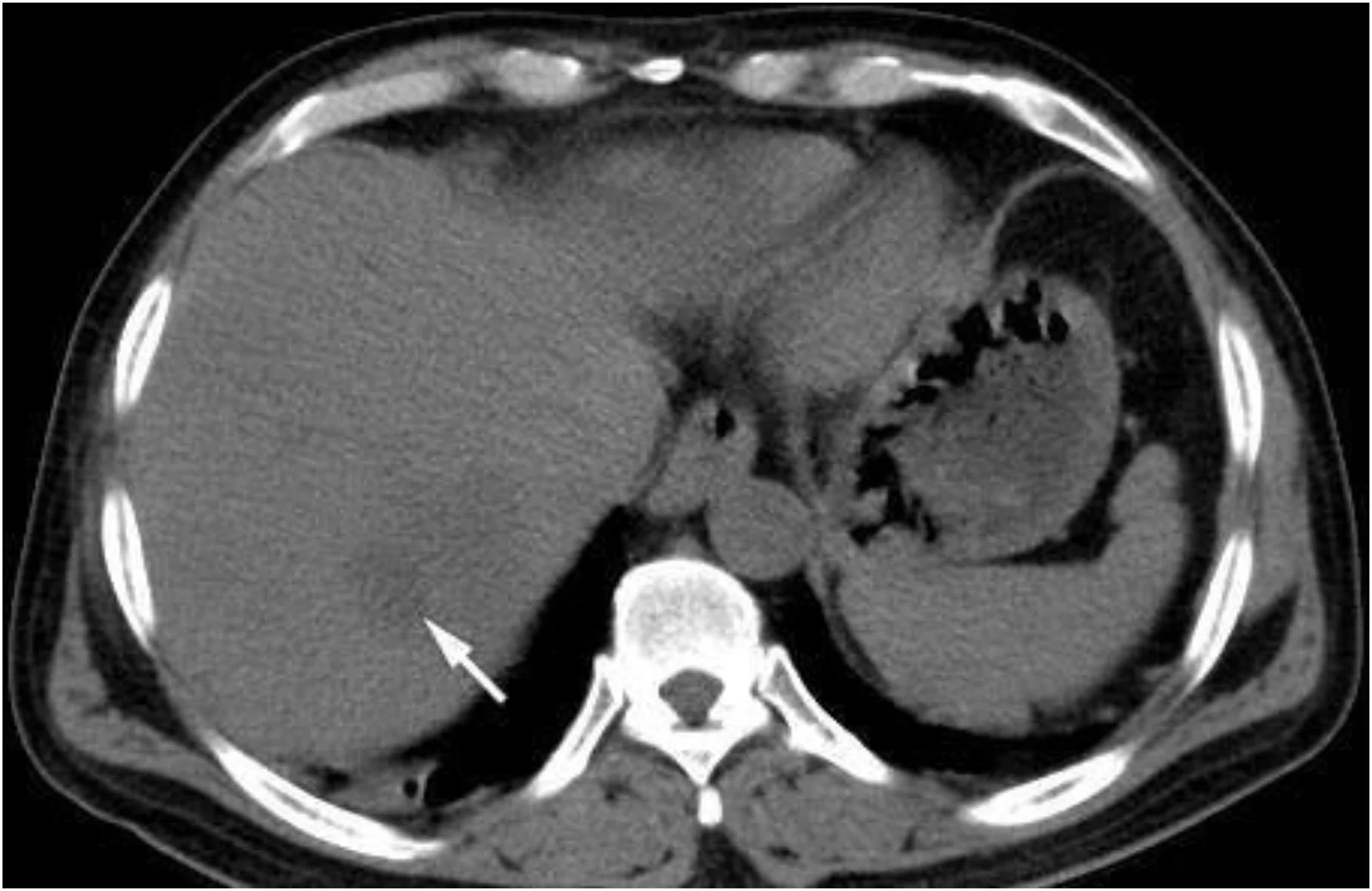
Axial noncontrast CT image demonstrating a well-defined hypoattenuating lesion in the right hepatic lobe, measuring approximately 20 HU (arrow).

**Fig. 2:**
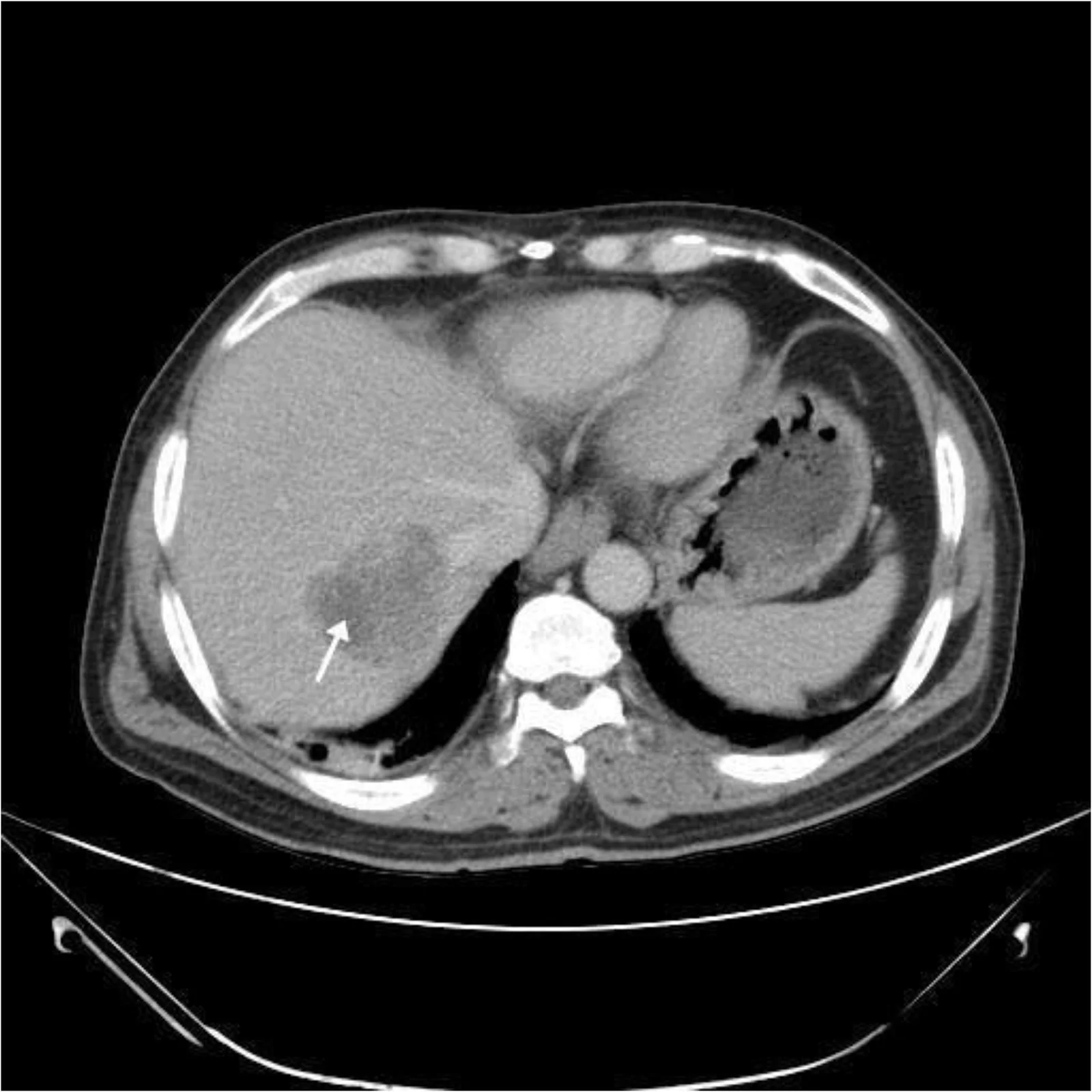
Axial portal venous-phase contrast-enhanced CT image demonstrating a segment VII hepatic lesion with thin, smooth peripheral rim enhancement and central low attenuation (arrow).

A segment VI lesion measuring approximately 3.2 × 3.5 cm was directly contiguous with the abnormal hepatic flexure, with focal discontinuity of the adjacent hepatic capsule and loss of the intervening fat plane (Fig. 3).

**Fig. 3:**
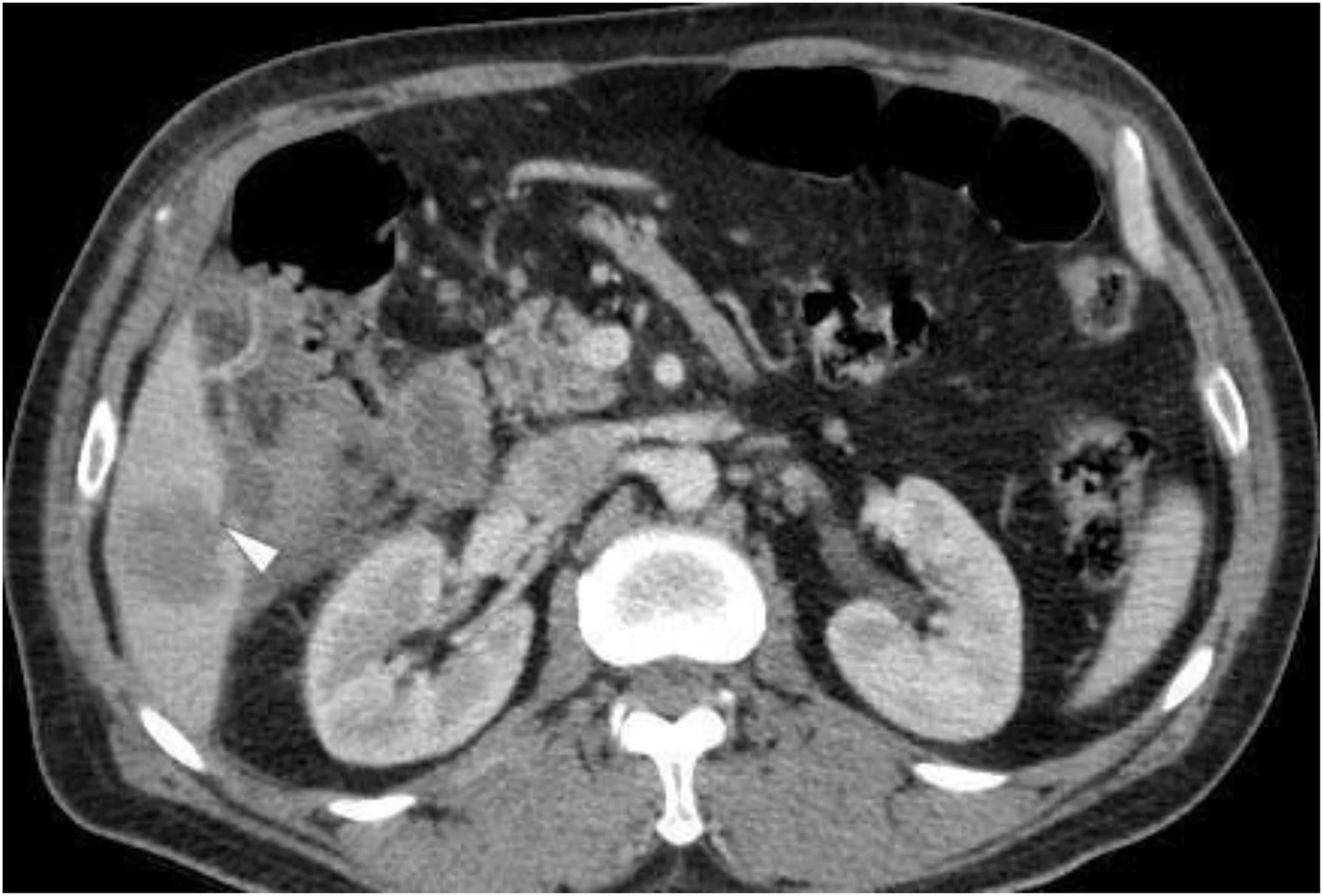
Axial portal venous-phase CT image demonstrating direct contiguity between the abnormal hepatic flexure and the adjacent segment VI hepatic lesion (arrowhead), with focal hepatic capsular discontinuity and loss of the intervening fat plane.

The hepatic flexure demonstrated irregular circumferential wall thickening measuring up to approximately 1.5 cm (Fig. 4A), with surrounding inflammatory fat stranding (Fig. 4B) and regional lymphadenopathy (Fig. 5). A right subcapsular/subhepatic collection was also present (Fig. 6A and B). No intralesional gas was identified.

**Fig. 4:**
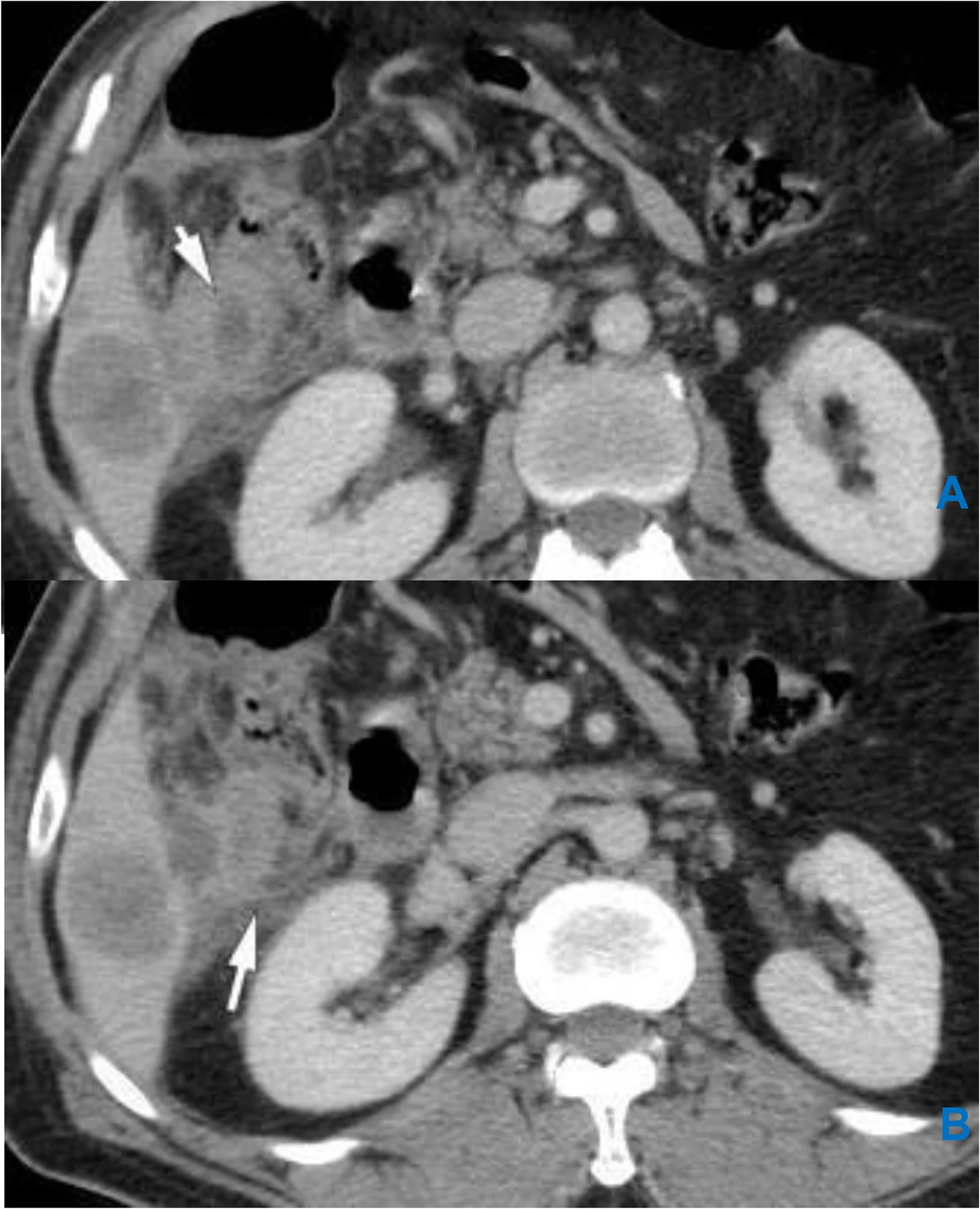
Portal venous-phase CT images demonstrating irregular circumferential wall thickening (arrow) of the hepatic flexure (A) and adjacent inflammatory fat stranding (B, arrow).

**Fig. 5:**
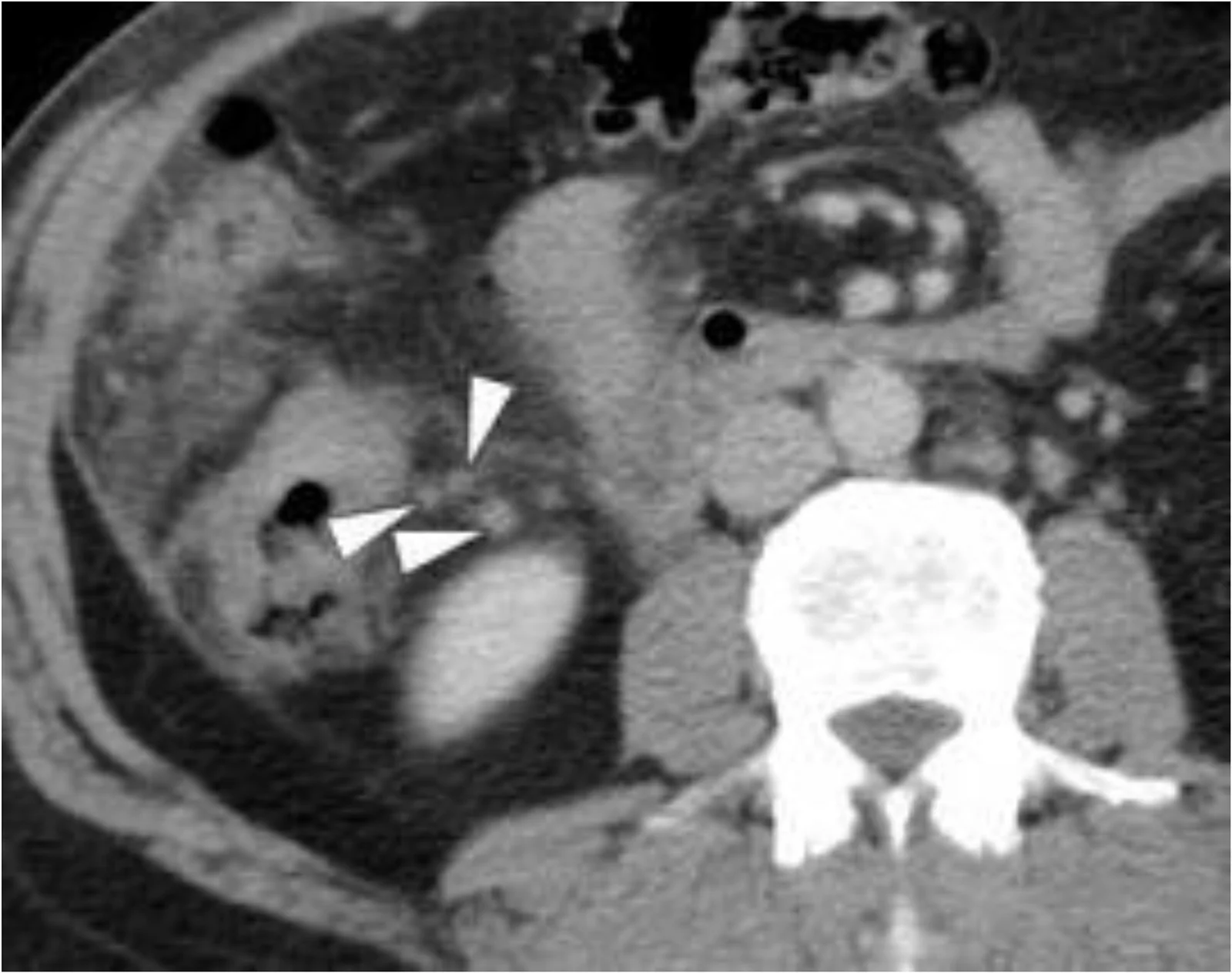
Portal venous-phase CT image demonstrating regional lymphadenopathy (arrowheads).

**Fig. 6:**
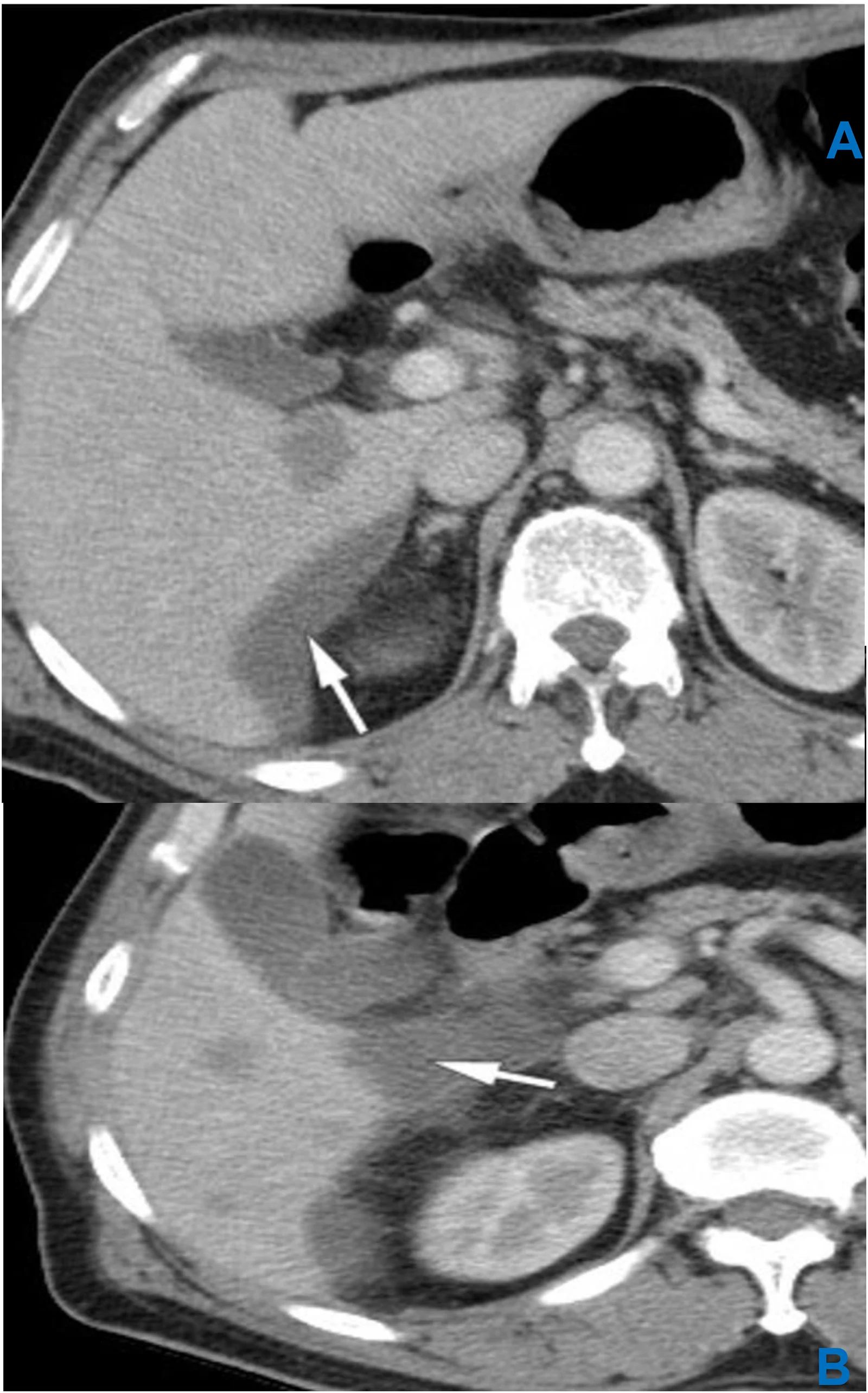
Arterial-phase contrast-enhanced CT images demonstrating a right subcapsular/subhepatic fluid collection (arrows) adjacent to the hepatic flexure on axial (A and B) views.

Although the irregular hepatic flexure thickening, regional lymphadenopathy, and multifocal hepatic lesions raised concern for colorectal malignancy with hepatic metastases, the low attenuation of the hepatic lesions, thin smooth peripheral enhancement, capsular disruption, adjacent inflammatory changes, and subcapsular/subhepatic collection created substantial overlap with pyogenic hepatic abscesses. The CT differential diagnosis therefore included both pyogenic hepatic abscesses and necrotic hepatic metastases associated with perforated hepatic flexure carcinoma. In the clinical setting of fever, chills, and inflammatory symptoms, the abscess-like interpretation was favored initially, and the patient was admitted with a working diagnosis of intra-abdominal sepsis secondary to a perforated viscus. Intravenous fluid resuscitation and broad-spectrum antibiotics were initiated, followed by emergency exploratory laparotomy.

Intraoperatively, a perforated hepatic flexure mass was identified, densely adherent to hepatic segments VI and VII (Fig. 7A), with fibrinopurulent exudate and inflammatory adhesions (Fig. 7B). An inflammatory tract between the hepatic flexure and adjacent liver parenchyma was noted, with associated hepatic inflammatory collections. Multiple nodular lesions were present in both hepatic lobes (Fig. 8A), along with para-aortic lymphadenopathy, peritoneal seeding (Fig. 8B), and approximately 500 mL of exudative fluid within Morrison’s pouch.

**Fig. 7:**
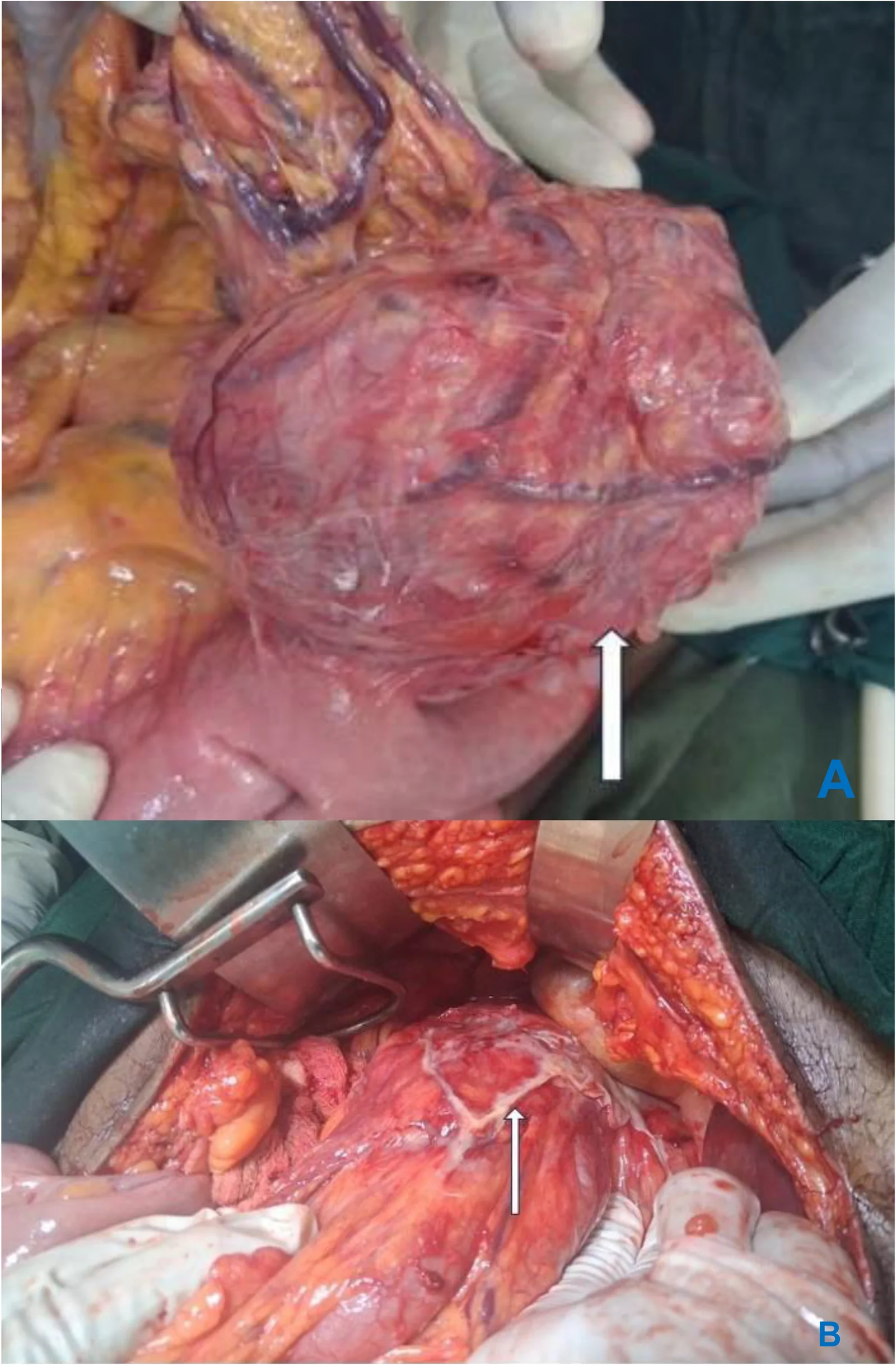
Intraoperative photographs demonstrating a perforated hepatic flexure mass adherent to the liver (A, arrow) and surrounding fibrinopurulent exudate (B, arrow).

**Fig. 8:**
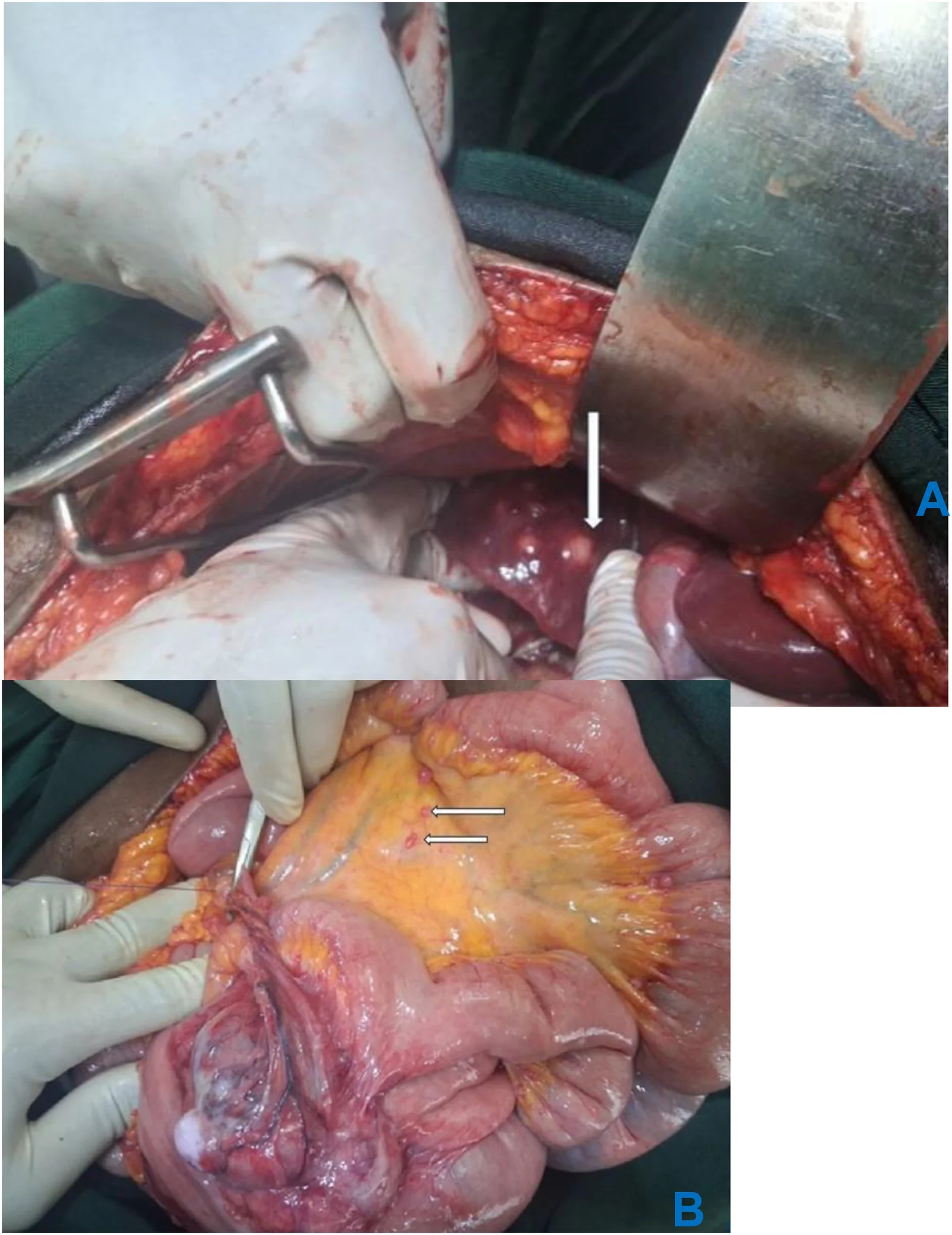
Intraoperative photographs demonstrating multiple hepatic nodular lesions (arrow) (A) and peritoneal tumor seeding (arrows) (B).

A right hemicolectomy with ileotransverse anastomosis was performed, and liver biopsy samples were obtained. The postoperative course was uncomplicated, and the patient was discharged on postoperative day 7. He was subsequently referred for oncologic management and completed 6 cycles of chemotherapy. At the latest available clinical follow-up, 8 months after surgery, he was reported to be clinically well.

Histopathologic examination of the colonic specimen confirmed a perforated moderately differentiated adenocarcinoma of the hepatic flexure (Fig. 9, Fig. 10). Liver biopsies demonstrated metastatic adenocarcinoma consistent with colorectal origin, with areas of central necrosis. Eight of fifteen lymph nodes were positive for metastasis, corresponding to stage pT4N2M1 according to the AJCC 8th edition.

**Fig. 9:**
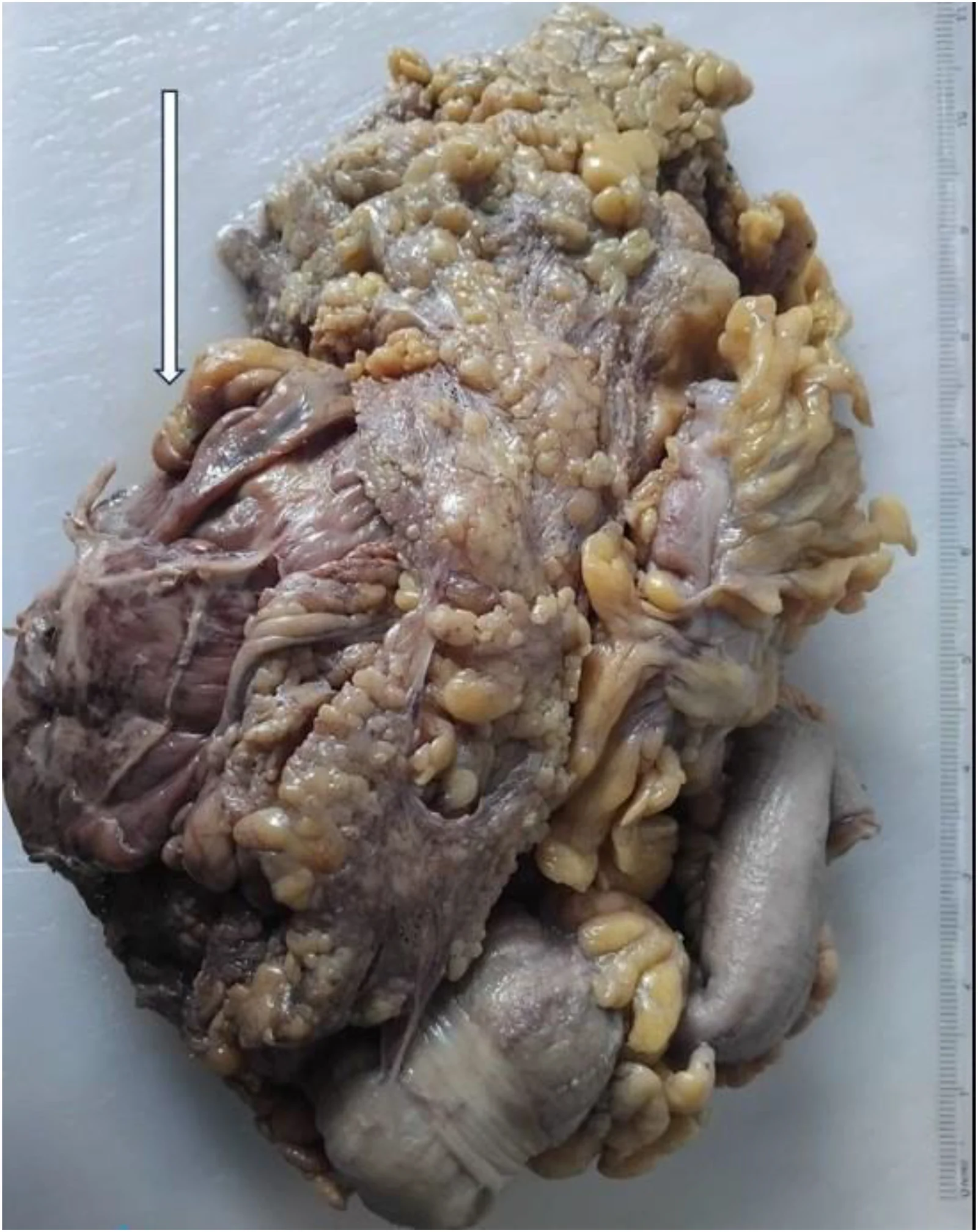
Gross pathologic specimen demonstrating the hepatic flexure tumor (arrow).

**Fig. 10:**
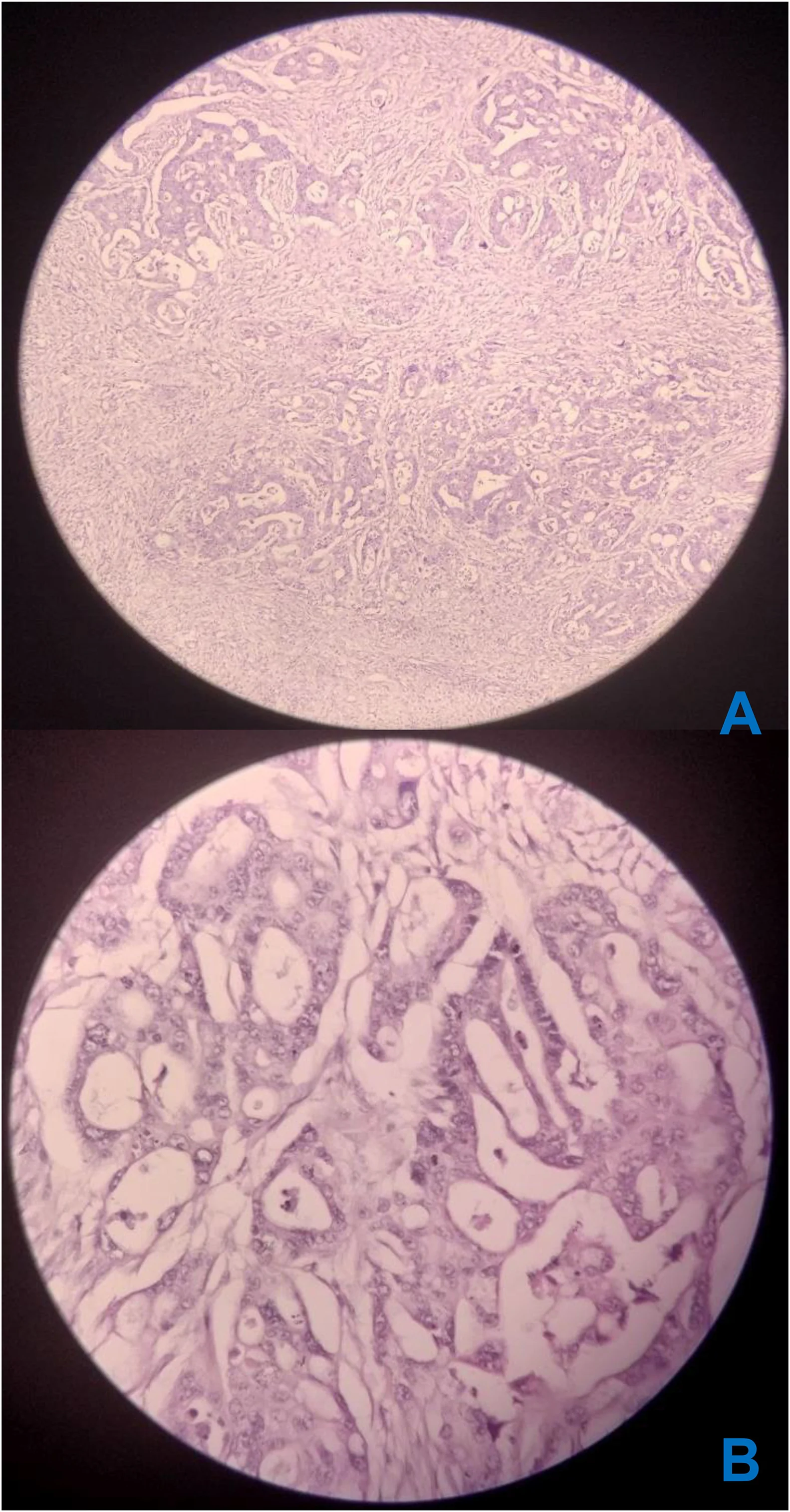
Histopathologic sections demonstrating moderately differentiated adenocarcinoma with gland formation at ×40 (A) and ×100 (B) magnification (hematoxylin and eosin stain).

## Discussion

Perforation is a recognized but relatively uncommon complication of colorectal carcinoma and may occur either freely into the peritoneal cavity or in a contained fashion adjacent to neighboring structures [11]. Tumors arising at the hepatic flexure are anatomically predisposed to involve the adjacent liver and subhepatic region because of their close proximity to hepatic segments V and VI [12,13]. Local tumor perforation may result in inflammatory phlegmon, direct hepatic invasion, or fistulous communication with adjacent hepatic tissue, thereby creating a complex radiologic appearance that may obscure the underlying malignancy [13]. In the present case, the perforated hepatic flexure tumor was densely adherent to the liver and associated with inflammatory changes and an apparent inflammatory tract to the adjacent hepatic parenchyma, creating an imaging appearance that closely resembled an infectious process.

The principal diagnostic challenge was distinguishing pyogenic hepatic abscesses from necrotic hepatic metastases. Several CT features favored an abscess-like process, including the low attenuation of the hepatic lesions, thin and smooth peripheral enhancement, focal hepatic capsular disruption, loss of the intervening fat plane between the hepatic flexure and adjacent liver, and the associated subcapsular/subhepatic collection and inflammatory fat stranding. In contrast, several features favored metastatic disease, including the multiplicity of hepatic lesions, involvement of multiple hepatic sites, irregular circumferential wall thickening of the hepatic flexure, and regional lymphadenopathy. The absence of intralesional gas also reduced the specificity of the abscess interpretation. Although classic CT findings of hepatic abscesses, such as the double-target sign, cluster sign, and intralesional gas, were not identified [8,9], the overall appearance still substantially overlapped with that of pyogenic abscesses.

Colorectal liver metastases may demonstrate central necrosis with low attenuation and peripheral enhancement, further contributing to this overlap [12,14]. No single imaging feature in this case reliably distinguished necrotic metastases from pyogenic abscesses. Instead, the combination of multifocal hepatic lesions, irregular colonic wall thickening, regional lymphadenopathy, and direct contiguity between the hepatic and colonic abnormalities should have maintained metastatic disease and perforation-related inflammatory extension as important differential considerations.

Direct hepatic invasion by perforated colonic carcinoma with associated inflammatory phlegmon represented a third important imaging consideration. CT findings supporting this diagnosis included focal discontinuity of the hepatic capsule, loss of the normal fat plane between the hepatic flexure and adjacent liver parenchyma, and direct contiguity between the colonic abnormality and a segment VI hepatic lesion. These findings raised suspicion for local tumor invasion and inflammatory communication, findings that were subsequently confirmed intraoperatively.

Pyogenic hepatic abscesses associated with occult colorectal carcinoma have been described in the literature, particularly in cases involving portal venous bacterial seeding [6,15]. However, direct tumor perforation with adjacent inflammatory extension producing an abscess-like hepatic appearance is less common [5,11]. In the present case, the hepatic lesions were confirmed histologically as metastatic adenocarcinoma with central necrosis, while surgery demonstrated fibrinopurulent exudate, inflammatory adhesions, and adjacent hepatic inflammatory collections. These findings suggest that the complex imaging appearance resulted from a combination of necrotic metastatic disease, localized tumor perforation, and secondary inflammatory reaction rather than isolated pyogenic hepatic abscess formation. The case therefore illustrates the substantial radiologic-pathologic overlap that can occur between necrotic metastases and inflammatory hepatic collections.

The patient's HIV infection also warrants consideration. In patients living with HIV, hepatic lesions may prompt early consideration of infectious etiologies, particularly when accompanied by fever, inflammatory changes, or abscess-like imaging features. This clinical context may increase the initial emphasis placed on infectious etiologies, particularly when necrotic tumors produce imaging findings that overlap with those of hepatic abscesses. However, the degree of immunosuppression in this patient cannot be established because recent CD4 count and viral load measurements were unavailable. Therefore, HIV infection should not be interpreted as evidence of significant immunocompromise in this case. The broader clinical context of HIV is nevertheless relevant because people living with HIV may have substantial non-AIDS comorbidities despite antiretroviral therapy, emphasizing the importance of maintaining a broad differential diagnosis rather than attributing new organ abnormalities solely to infection [10]. A recent multicenter study from Ethiopia reported a substantial burden of cardiovascular abnormalities among adults living with HIV, illustrating the broader spectrum of non-AIDS comorbidity in this population [16]. In the present case, the combination of HIV infection, systemic inflammatory symptoms, and abscess-like hepatic lesions may have increased the plausibility of an infectious diagnosis, but the concurrent irregular hepatic flexure thickening, regional lymphadenopathy, and multifocal hepatic lesions were important features that should have maintained malignancy in the differential.

This case highlights several important radiologic considerations. First, inflammatory-appearing colonic wall thickening should not exclude malignancy, particularly when accompanied by regional lymphadenopathy or adjacent hepatic abnormalities. Second, hepatic lesions contiguous with an abnormal colonic segment warrant careful assessment for direct invasion, metastatic disease, or perforation-related inflammatory extension. Finally, perforated colorectal carcinoma should remain an important differential diagnosis in patients presenting with apparent pyogenic hepatic abscesses, especially when imaging findings are multifocal or associated with suspicious colonic abnormalities. In the present case, the final diagnosis required operative and histopathologic correlation due to overlapping CT features between necrotic metastases and pyogenic hepatic abscesses, underscoring the limitations of imaging in differentiating complex inflammatory lesions from metastatic disease. This case illustrates an uncommon diagnostic presentation of perforated hepatic flexure adenocarcinoma mimicking multiple pyogenic hepatic abscesses.

## Conclusion

Perforated hepatic flexure adenocarcinoma with necrotic hepatic metastases may closely mimic pyogenic hepatic abscesses on imaging. When abscess-like hepatic lesions occur alongside suspicious colonic abnormalities or regional lymphadenopathy, malignancy should remain an important diagnostic consideration. The case therefore illustrates the substantial radiologic-pathologic overlap between necrotic metastases and inflammatory hepatic collections.

## Learning points

1. Necrotic hepatic metastases may closely mimic pyogenic hepatic abscesses, particularly when associated with inflammatory changes from perforated colorectal carcinoma.
2. When abscess-like hepatic lesions coexist with irregular colonic wall thickening, regional lymphadenopathy, or other suspicious features, colorectal malignancy should remain an important differential diagnosis.
3. In cases with substantial overlap between inflammatory and malignant imaging features, correlation with operative findings and histopathology may be essential for definitive diagnosis.

## Data availability

All relevant data supporting the findings of this study are included within the article.

## Author contributions

All authors contributed to patient management, data collection, manuscript preparation, critical revision of the manuscript, and approval of the final version.

## Patient consent

Written informed consent was obtained from the patient for publication of this case report and the accompanying images.
